# Role of T-Helper 9 Cells in Chronic Hepatitis C-Infected Patients

**DOI:** 10.3390/v10070341

**Published:** 2018-06-24

**Authors:** Mohamed E. Ali, Omnia El-Badawy, Noha A. Afifi, Abeer Sharaf Eldin, Elham Ahmed Hassan, Hamada M. Halby, Mohamed Ahmed El-Mokhtar

**Affiliations:** 1 Department of Microbiology and Immunology, Faculty of Pharmacy, Al-Azhar University, Assiut 71524, Egypt; elkady4work@azhar.edu.eg (M.E.A.); hamadahalby@ymail.com (H.M.H.); 2 Department of Medical Microbiology and Immunology, Faculty of Medicine, Assiut University, Assiut 71515, Egypt; omniaalbadawy@aun.edu.eg (O.E.-B.); noafifi2000@yahoo.com (N.A.A.); 3 Department of Gastroenterology and Tropical Medicine, Faculty of Medicine, Assiut University, Assiut 71515, Egypt; abeer.ahmed@aun.edu.eg (A.S.E.); elham.abdelhalem@aun.edu.eg (E.A.H.)

**Keywords:** hepatitis C virus, T-helper 9 cells, HCV-related complications

## Abstract

Hepatitis C virus is a hepatotropic virus that is transmitted parenterally. Viral infections are usually associated with modulations of the immune cells, leading to enhanced viral survival and spreading, and accordingly, life-threatening complications. Recently, it has been proposed that a new subset of T-helper, named T-helper 9, is involved in the pathogenesis of different immunopathological conditions, such as allergies, tumors, and viral infections. Some studies reported a protective role, and others described a pathogenic potential for the T-helper 9 cells. Here, we present evidence that T-helper 9 cells are dynamically increased with increasing the pathogenic strategy for hepatitis C virus (HCV). Furthermore, viral clearance is associated with a decrease in T-helper 9. The increase in T-helper 9 was paralleled with an increase in its receptor expression. Taken together, our data suggest that T-helper 9 cells play an important role in the pathogenesis of HCV, and is directly associated with HCV-related complications.

## 1. Introduction

Hepatitis C virus (HCV) is a small (55–65 nm in size), enveloped, positive-sense single-stranded RNA virus of the family *Flaviviridae* [1]. About 71 million people are chronically infected with HCV worldwide. Egypt has the highest prevalence of HCV in the world. Nearly 15 million Egyptians currently suffer from HCV, with 40,000 dying from the disease each year [2]. Genotype 4 is the predominant genotype, which accounts for more than 90% of infections [3,4].

Following the natural course of infection, 20–30% of infected patients develop liver cirrhosis within 20–30 years after infection [5]. Of these patients, 1–8% will develop hepatocellular carcinoma (HCC), and 67–91% will die due to liver-related causes [6]. HCV infection causes approximately one-third of all HCC cases globally [7]. In Egypt, the relative frequency of liver-related cancers has increased from 4% to 7.3%, with HCV accounting for 40–50% of cases, which underlines the important role of HCV in the pathogenesis of liver-related complications [8].

Treatment of HCV infections with directly acting antiviral agents (DAAs) has provided a promising role in the eradication of this infection. Second-generation and third-generation DAA combinations can provide a promise to cure >95% of patients in as little as 6 weeks of treatment [9,10].

CD4^+^ T-helper (Th) cells are principle modulators of the immune response. Upon activation, distinct Th subsets are generated to perform specialized functions [11]. T-helper 9 (Th9) cells constitute a recently defined subset of Th cells that secrete IL9. These cells develop under the influence of both transforming growth factor and IL4, and are controlled by interferon regulatory factor (IRF)-4, and STAT-6 [12,13].

Several studies have described controversial protective and pathogenic roles for Th9 cells in different diseases. Th9 cells are major contributors to asthma, food allergies, ulcerative colitis, and multiple sclerosis [14]. On the other hand, Th9 cells promote the immune response against helminthes. It has been shown that Th9 cells are predominantly involved in both pathogen clearance and immune response activation against parasites. Moreover, Th9 cells mediate important antitumor immune responses, particularly in cases of melanoma [15,16].

A recent study has shown that Th9 cells were involved in the pathogenesis of hepatitis B virus (HBV) and the development of hepatic fibrosis [17]. However, as far as we know, the role of Th9 cells in the immune pathogenesis of HCV-related chronic liver disease has not been investigated yet. Furthermore, little is known about the changes in the frequency of Th9 cells associated with antiviral therapy, particularly in the era of direct-acting anti-HCV therapy. Therefore, in the present study, we aimed to assess the potential association between the frequency of Th9 cells and the clinical characteristics in different groups of HCV-infected patients, to provide a better understanding of the immunopathological role of Th9 cells in HCV-related diseases.

## 2. Materials and Methods

### 2.1. Ethics Statement

This work has been approved by the Local Ethical Committee of the Faculty of Medicine, Assiut University. All participants were adults, and all of them provided written informed consent before collection of samples.

### 2.2. Study Subjects

This study was carried out at the Medical Research center, Faculty of Medicine, Assiut University, Egypt from December 2016 to December 2017. The study protocol was approved by the local ethical committee. Informed consent was obtained from all subjects.

Eighty-five chronic HCV-infected patients and twenty sex- and age-matched healthy controls were enrolled in the study. Subjects were recruited from patients attending the Gastroenterology and Tropical Medicine Department, and AL-Rajhi Liver hospital, Egypt, and were divided into three groups: 16 patients with chronic HCV infection (CHC), 43 patients with liver cirrhosis (LC), and 26 patients with LC and hepatocellular carcinoma (HCC).

CHC patients were diagnosed by laboratory findings of fluctuations of serum transaminases levels for more than 6 months, positive HCV antibodies and serum HCV-RNA with the absence of sonographic findings of liver cirrhosis. Diagnosis of LC was based on clinical, biochemical, and ultrasonographic findings. The severity of liver cirrhosis was assessed according to Child–Pugh and MELD scores [18,19]. Furthermore, cirrhotic patients were divided according to the hepatic focal lesion(s), elevated alpha fetoprotein-L3 (AFP-L3), and imaging.

Treatment of patients was based on direct antiviral agents (DAAs). Patients received sofosbuvir ± ribavirin therapy. Sustained virological response (SVR) was defined as undetectable HCV-RNA in serum after six months of treatment.

We excluded patients who were treated with interferon, ribavirin, or immunomodulating agents, co-infected with hepatitis A, B viruses or human immunodeficiency virus, those having alcohol or drug-induced liver diseases, or those with positive anti-schistosomal antibodies.

### 2.3. Sample Processing

Blood samples were collected from all study subjects. Samples were collected into heparinized tubes labeled with the patient’s name, sex, age, and the date of collection. The peripheral blood mononuclear cells (PBMCs) were separated by Ficoll density gradient centrifugation (Biowest, Riverside, MO, USA). Part of the isolated PBMCs was used for estimating the level of CD4^+^ IL9^+^ T cells by flow cytometry, and another part was used for RNA extraction to be used in quantitative real-time PCR (qRT-PCR) for estimating the level of *IL9R* expression.

### 2.4. Cell Preparation

PBMCs were suspended at a density of 2 × 10^6^ cells/mL in complete culture medium (RPMI 1640 supplemented with 100 U/mL penicillin, 100 µg/mL streptomycin, 2 mM glutamine, and 10% heat-inactivated fetal calf serum (Gibco, Waltham, MA, USA) in a 6-well plate. Cells were stimulated with 25 ng/mL phorbol myristate acetate (PMA) plus ionomycin (1 µg/mL) (Biovision, Milpitas, CA, USA) in the presence of brefeldin A (BioLegend, San Diego, CA, USA). Cells were incubated at 37 °C in presence of 5% CO_2_ for 6 h, then analyzed by flow cytometer.

### 2.5. Flow Cytometry Analysis

**Figure 1:**
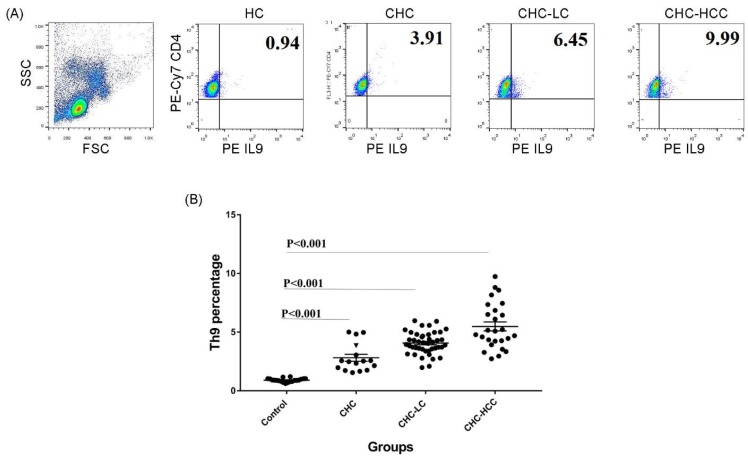
Frequency of Th9 cells in different patient groups. (**A**) Gating strategy and representative histograms used to determine the Th9 subset in different patient groups. (**B**) Summary of Th9 percentages in different study groups. CHC: chronic hepatitis C patients, CHC-LC: chronic hepatitis C associated liver cirrhosis, CHC-HCC: chronic hepatitis C associated hepatocellular carcinoma, HC: healthy controls.

Flow cytometry was used for analyzing the CD4^+^ IL9^+^ T subset. Cells were stained with PE-Cy7 anti-human CD4 (eBioscience, San Diego, CA, USA) in a dark place at room temperature for 20 min. After surface staining, cells were fixed, permeabilized and then stained with phycoerythrin (PE) anti-human IL9 (eBioscience, San Diego, CA, USA). To determine cut-offs, matching isotype control antibodies were processed in a similar way. In this study, CD4^+^ IL9^+^ T cells were referred to as the Th9 cells. Cells were calculated as a percentage of CD4^+^ T cells. Stained cells were acquired by FACS-Caliber flow cytometer (BD Bioscience, San Jose, CA, USA) and data were analyzed using FlowJo software 7.6.1 (Tree Star Inc., Ashland, OR, USA). The gating strategy is demonstrated in Figure 1.

### 2.6. Real-Time PCR

Total RNA was extracted from PBMCs using TRIZOL reagent (Invitrogen, Carlsbad, CA, USA), and then reverse transcribed into cDNA with Reverse Transcription kit, according to the manufacturer’s instructions (Intron, Biotechnology, Seoul, South Korea). Real-time PCR was performed using SYBR Green (Bioline, Taunton MA, USA) and ABI 7500 Sequence Detection System (Applied Biosystems, Foster City, CA, USA). After an initial denaturation step for 5 min at 94 °C, a three-step cycling procedure (denaturation at 94 °C for 30 s, annealing at 60 °C for 30 s, and extension at 72 °C for 60 s) was used for 40 cycles. The primer sets specific for transcripts of genes were as follows:

Forward (5′CGTGCCCTCTCCAGCGATGTTCT3′) and reverse (5′GACGCGCTGGGCCACAAGTG3′) for IL9R gene [20]. Forward (5′GGATTTGGTCGTATTGGG3′) and reverse (5′GGAAGATGGTGATGGGATT3′) for GAPDH as a housekeeping gene [21]. *IL9R* expression level was normalized to the level of GAPDH transcripts and quantified by the 2^−ΔΔ*C*T^ method.

### 2.7. Statistical Analysis

Data were expressed as the mean ± standard deviation (SD). Two group comparisons were carried out using Student’s *t*-test or Mann–Whitney U test when appropriate. One-way ANOVA test was used for multiple comparisons. Correlations were determined by Pearson’s correlation test. All data were analyzed using SPSS 16.0 software (SPSS Inc., Chicago, IL, USA). *p* values of less than 0.05 were considered as statistically significant.

## 3. Results

### 3.1. Alteration of the Frequency of Th9 Cells in Infected Patients

The demographic and clinical characteristics of study populations are demonstrated in Table 1. The percentage of the circulating CD4^+^ IL9^+^ T subset in different groups of HCV-infected patients was analyzed determined. Gating strategies for identifying the CD4^+^ IL9^+^ T-cell population are shown in Figure 1A. Generally, percentages of Th9 cells in different patient groups were significantly higher than that of healthy controls (HC), and there were significant differences between the CHC, CHC-LC, and CHC-HCC groups (Figure 1B). The mean percentage of Th9 cells in the peripheral blood of CHC patients (2.9% ± 1.3) was clearly elevated compared with the healthy control group (0.9% ± 0.2; *p* < 0.001). When patients were divided into subgroups according to the clinical complications, the percentage of Th9 cells presented a dynamic increase in both CHC-LC and CHC-HCC groups compared to healthy controls (4% ± 0.9 and 5.5% ± 1.9, respectively, vs 0.9% ± 0.2) as shown in Figure 1B.

### 3.2. Correlation of Th9 Cells with Clinical Parameters

**Figure 2:**
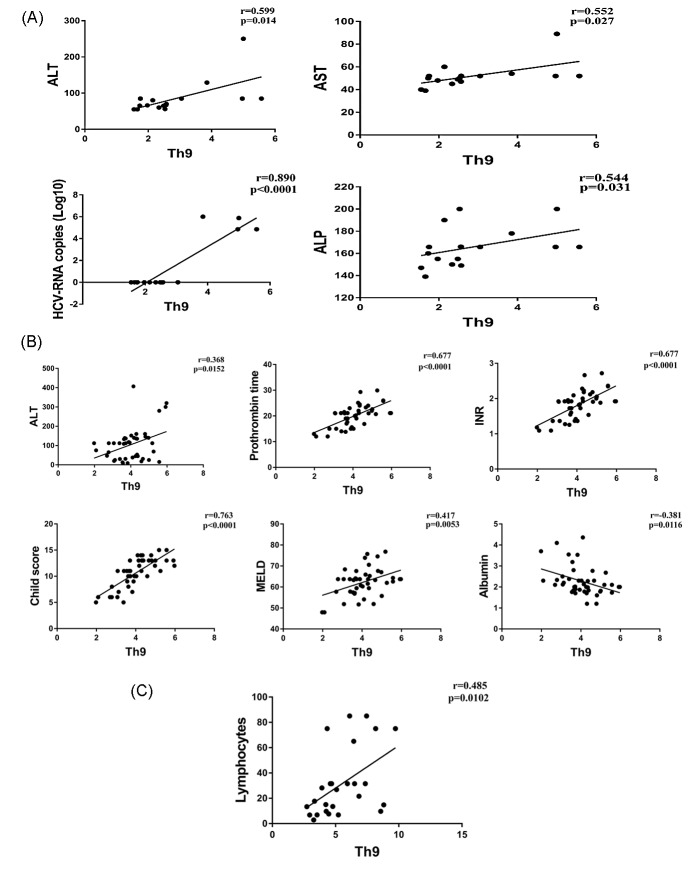
Correlations of Th9 percentage and laboratory parameters in CHC (**A**); CHC-LC (**B**); and CHC-HCC patients (**C**).

Correlations of Th9 percentage with the clinical parameters of each patient group were analyzed. The percentage of Th9 cells in CHC patients was positively correlated with the levels of liver enzymes ALT (*r* = 0.599, *p* = 0.014), AST (*r* = 0.552, *p* = 0.027), ALP (*r* = 0.544, *p* = 0.031), and HCV-RNA copy number (*r* = 0.890, *p* < 0.0001). For the CHC-LC patient, Th9 cells were positively correlated with ALT (*r* = 0.368, *p* = 0.015), prothrombin time (*r* = 0.677, *p* < 0.0001), INR level (*r* = 0.677, *p* < 0.0001), Child**–**Pugh and MELD scores (*r* = 0.763, *p* < 0.0001 and *r* = 0.417, *p* = 0.005 respectively). In addition, a negative correlation was observed with serum albumin (*r* = 0.381, *p* = 0.011). However, only the lymphocyte count was positively correlated with Th9 cells in the HCC patients (*r* = 0.485, *p* = 0.01) (Figure 2A–C).

### 3.3. Th9 Levels in Relation to Liver Disease Severity

**Figure 3:**
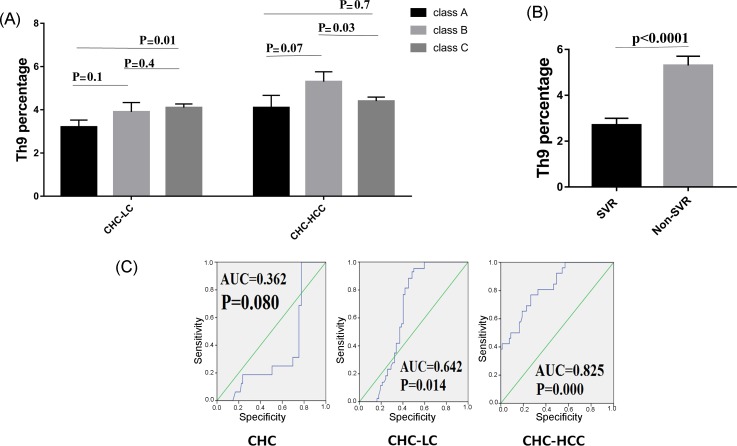
Correlations of Th9 percentage and patient characteristics. (**A**) Th9 percentage in correlation to severity of liver disease. (**B**) Lower levels of Th9 cells were observed in patients who achieved sustained virological response (SVR). (**C**) Receiver operating characteristic curve of Th9 for the prognosis prediction in chronic hepatitis C infected patients.

We further analyzed the possible association between Th9 cells and the severity of liver cirrhosis assessed by the Child**–**Pugh score. In the CHC-LC group, patients with Child**–**Pugh class C had a significantly higher Th9 percentage (4.1% ± 0.9) than those with Child**–**Pugh class A (3.2% ± 0.8, *p* = 0.01). However, for CHC-HCC group, a significant difference was observed between classes B and C (5.3% ± 2 vs. 4.4% ± 1.2, *p* = 0.03), as shown in Figure 3A.

### 3.4. Th9 Levels in Relation to Response to Treatment

HCV patients were divided into two groups to determine if there was a possible association between Th9 cells and successful clearance of HCV. Interestingly, Th9 level was lower in the patients who achieved a sustained virological response (SVR) compared to those who did not achieve SVR (2.7% ± 1.3 vs 5.3% ± 1.4, *p* < 0.0001), as shown in Figure 3B.

### 3.5. Predictive Accuracy and Determination of the Best Cut-Off Value of Th9 Cells for Predicting HCV-Related Chronic Liver Disease

ROC curves for circulating Th9 cells for prediction of CHC, LC with and without HCC, showed better prognostic accuracy for prediction of LC with HCC (AUC = 0.825; 95% CI, 0.739–0.893). The cut-off deriving from ROCs with the best ability to predict patients with HCC was >4.18 with a sensitivity of 77%, specificity of 73.4%, and net present value (NPV) of 87.8%. However, for prediction of LC without HCC, the cut-off value of >3.05 had 88.4% sensitivity, 54.8% specificity, and 82.2% NPV. Furthermore, the cut-off value of >2.5 with 68.8% sensitivity, 75.3% specificity, and 91.2% NPV for prediction of CHC, as shown in Figure 3C.

### 3.6. Expression of IL9 Receptor (IL9R) in HCV-Infected Patients

**Figure 4:**
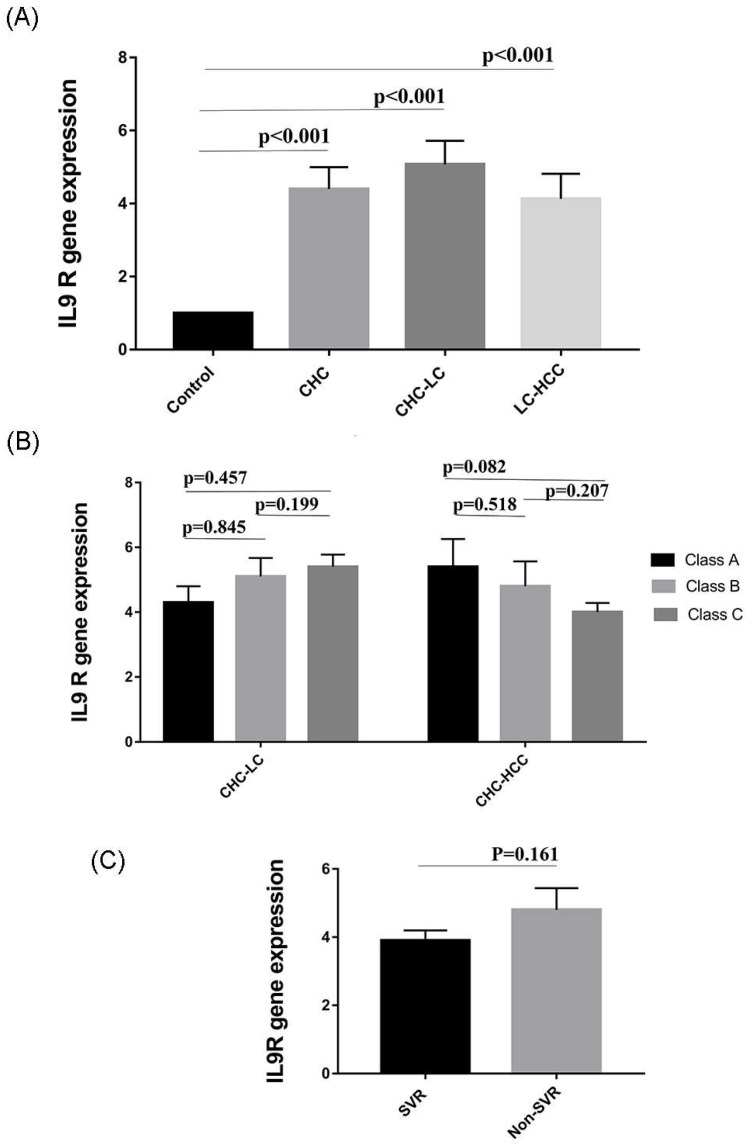
Variation in *IL9R* expression in different study populations. (**A**) *IL9R* expression is elevated in HCV-infected patients. (**B**) *IL9R* expression in relation to Child–Pugh score as a measure of the severity of liver damage. (**C**) *IL9R* expression in patients who received directly acting antiviral agents (DAAs).

We wondered if changes in Th9 levels were associated with parallel changes in *IL9R* expression. Therefore, the expression of *IL9R* in PBMCs of patients was analyzed by qRT-PCR. Interestingly, gene expression of IL9R was significantly elevated in the 3 patient groups (CHC, CHC-LC, and CHC-HCC) compared to healthy controls (*p* values = 0.006, <0.001, <0.01 respectively). The mean *IL9R* expression in LC-HCC patients was lower than CHC and CHC-LC patients, but this difference, however, was not statistically significant (Figure 4A). There was no obvious correlation between *IL9R* expression and Child–Pugh score, as shown in Figure 4B. Similarly, no difference was observed in *IL9R* expression in patients who achieved sustained virological response (SVR) compared to non SVR patients, as shown in Figure 4C. In addition, no significant correlations were detected between *IL9R* expression and patients’ laboratory parameters in all groups as shown in Table 2.

## 4. Discussion

The association between Th9 cells and multiple human diseases is rapidly developing. The contribution of Th9 cells in determining the outcome of different immunopathological conditions, such as the development of asthma, allergic rhinitis, ulcerative colitis, SLE, helminthes infections, and melanoma has been described [14,22,23]. Previous studies demonstrated the role of Th subsets and their cytokines, such as Th1/IFN-γ, Th2/IL4, Treg/IL10, and Th17/IL17 in the regulation of HCV infection [24,25,26,27]. However, the role of Th9/IL9 in the progression of HCV infections has not been reported until the present moment. In this study, we analyzed the percentages of Th9 cells in different stages of HCV infection. We observed an obvious relation between Th9 cell frequency and HCV-related disease severity. Our results also showed that Th9 percentage was positively correlated with clinical parameters such as liver enzymes, HCV copy number, and response to antiviral therapy or Child–Pugh and MELD scores, according to patient group.

Our findings suggest that Th9 cells in HCV-infected patients are directly linked to the progression and prognosis of the liver disease. Several studies have also described the association between Th9/IL9 and viral infections. For example, Zika virus infection was associated with an increased secretion of IL2, IL4, IL13, and IL9 cytokines [28]. Severe infection with the acute respiratory syncytial virus was associated with elevated IL9 levels [29]. Moreover, HCV induced the release of different Th1 cytokines (MIP-1α, IP-10, TNFα, IL12p70, and IL2), Th17 cytokines (IL6, IL8, IL9, and IL17) and profibrotic factors (EGF-b, VEGF). The authors also reported that treatment with PEGylated interferon-alpha plus ribavirin was associated with downregulation of the secretion of Th1, Th17 proinflammatory mediators, and pro-fibrotic growth factors in responder patients, especially in MIP-1α, FGF-b, and IL17 [30]. Similarly, [31] showed that IL6 and IL9 elevations were predictive for HCV treatment failure, while HCV clearance was associated with low inflammatory and Th9 cytokine levels [31].

The elevation of Th9 cells in complicated HCV patients could be mediated by its effect on Th17 expansion, which leads to significant liver inflammation and damage [27,32,33,34,35,36]. Another explanation is that IL9 inhibits Th1 immune responses, which leads to persistence of viral infection [24,37,38]. Moreover, it has been shown that Th9 cells enhance the suppressive function of regulatory FOXP3^+^ CD4^+^ cells [39].

Furthermore, HCC patients with higher tumor-infiltrating Th9 frequency had significantly shorter disease-free survival period after resection [40]. The frequency of Th9 and Th17 cells was distinctly elevated in patients with stage III or IV cancers as compared with those in stage I or II. Patients with distant metastasis or lymph node metastasis also showed a higher frequency of Th9 and Th17 cells than those without metastasis [41].

Based on these observations, it could be concluded that the persistence of HCV and associated viral antigens induced a higher inflammation response through Th9 cells, which also explains the elevation of Th9 cells in non-SVR patients [42]. Supporting these findings, IL9 neutralization reduced the development of hepatic inflammation, necrosis, and fibrosis, and anti-IL9Ab treatment markedly decreased the number of activated hepatic stellate cells [17].

Interestingly, the correlation of Th9 levels with progression of chronic HBV infections shows a lot of discrepancies. Sun, et al. [43] reported a higher frequency of circulating Th9 cells in chronic HBV patients. The percentage of Th9 cells was also positively correlated with ALT level. By contrast, another group of investigators reported there was no significant difference in the percentage of Th9 cells, serum IL9 and IL10 levels among different groups (CHB and CHB-LC), no change in the percentage of Th9 cells before and after antiviral treatment in CHB patients, and no obvious correlation between the percentage of Th9 cells, levels of IL9, and IL10 with age, total bilirubin, albumin, ALT, and HBV DNA levels [44]. Similarly, Cui, et al. [45] showed that the frequency of circulating Th9 cells was significantly lower in chronic HBV patients than controls, and the percentages of Th9 cells were negatively correlated with HBV DNA loads. The discrepancies might be explained, at least in part, to the difference in levels of HBV DNA load and the difference in study populations.

Th9 cells were associated with HCV-related inflammation. To investigate whether changes in IL9 were associated with alterations in its cognate receptor IL9R, we analyzed the change in *IL9R* mRNA in PBMCs of all study subjects by qRT-PCR. Expression of *IL9R* was significantly elevated in the three patient groups (CHC, CHC-LC, and LC-HCC). In agreement with our data, IL9R has been associated with different inflammatory, autoimmune, and malignant diseases, such as psoriasis lesion, lupus erythematous, and various human leukemias, including T-cell leukemia, megakaryoblastic leukemia, and Hodgkin lymphomas [23,46,47,48]. *IL9R* overexpression in HCC cells inhibited cell apoptosis, promoted cell proliferation, and increased their invasive potential, possibly through the involvement of VEGF, p-p38, p-STAT3, and MMP9 proteins. In addition, IL9R was reported to be an independent predictor for HCC patients [21,48,49,50].

This study provided novel information about the role played by Th9 in the pathogenesis of HCV infections. Based on our observations, the level of Th9 cells correlated with liver disease activity and severity and may, therefore, represent a useful biomarker for HCV-related complications. Our results underline the potential use of Th9/IL9 based immunotherapy as a promising approach for treating HCV-related liver diseases.

## Figures and Tables

**Table 1:** Demographic, clinical, and laboratory characteristics of the study patients.

Variable	CHC (*n* = 16)	LC without HCC (*n* = 43)	LC with HCC (*n* = 26)	Total (*n* = 85)
Age (years) ^a^	47 ± 13.4 (25–65)	59.9 ± 8.4 (45–78)	58.3 ± 9.4 (41–80)	57 ± 10.7 (25–80)
Sex ^b^				
Male	8 (50%)	24 (55.8%)	14 (53.8%)	46 (54.1%)
Female	8 (50%)	19 (44.2%)	12 (46.2%)	39 (45.9%)
Co-morbid diseases ^b^	2 (13%)	11 (26%)	12 (46%)	25 (29.4%)
Abdominal pain ^b^	5 (31%)	6 (14%)	13 (50%)	24 (28.2%)
Ascites ^b^	0	32 (74%)	15 (58%)	47 (81%)
GIT bleeding ^b^	0	8 (19%)	8 (31%)	16 (18.8%)
Hepatic Encephalopathy ^b^	0	25 (58%)	10 (38%)	35 (41%)
Serum albumin ^a^	4.1 ± 0.7	2.3 ± 0.7	2.6 ± 0.7	2.7 ± 0.9
Serum total bilirubin (gm/dL) ^c^	1.8 (1.2–4)	3.1 (0.4–7.2)	1.9 (0.8–24.1)	2 (0.4–24.1)
Alanine transaminase (IU/L) ^c^	68.5 (55–250)	112.2 (8–407)	100.1 (22–310)	84.8 (8–407)
Aspartate transaminase (IU/L) ^c^	51 (39–89)	71 (14–222)	92 (32–476.3)	66 (14–476.3)
Alkaline phosphatase (IU/L) ^c^	165.8 (139–200)	168 (55–420)	179 (57–340)	166 (55–420)
Prothrombin time (seconds) ^a^	14 ± 1.5	19.5 ± 4.4	20.5 ± 10.5	19.8 ± 7.7
INR ^a^	1.1 ± 0.3	1.7 ± 0.4	1.9 ± 0.9	1.7 ± 0.7
WBC(x10^6^/uL) ^c^	4.3 (2.4–5.2)	6.1 (2.4–33.5)	8.5 (1.5–31.8)	7.5 (1.5–33.5)
Lymphocytes ^c^	15.2 (11.3–76)	19.6 (6.1–75)	15 (2.9–85)	17.7 (2.9–85)
Neutrophils ^c^	70.1 (55–78)	71.2 (52.2–83.2)	76 (53.4–89.5)	73.6 (52.2–89.5)
Hematocrit value ^c^	31 (22–35)	30 (11.7–43.5)	28.9 (13.5–49.1)	29.6 ± 7.7
Serum creatinine (mg/dL) ^c^	1.1 (0.8–1.3)	1.4 (0.5–4)	1.2 (0.5–5.9)	1.2 (0.5–5.9)
Alfa Fetoprotein (IU/L) ^c^	NA	NA	357 (61.6–2526.6)	357 (61.6–2526.6)
Child–Pugh score ^a^	0	10.7 ± 2.7	10.2 ± 2.8	10.5 ± 2.7
Child–Pugh classification ^b^				
Child–Pugh A	NA	6 (14%)	3 (12%)	9 (8.9%)
Child–Pugh B	NA	9 (21%)	10 (38%)	19 (18.1%)
Child–Pugh C	NA	28 (65%)	13 (50%)	41 (39%)
MELD score ^a^	0	17.7 ± 7.1	19.5 ± 8.9	18.4 ± 7.8
Death ^b^	0	1 (2.3%)	6 (23%)	7 (8.2%)

CHC: patients with chronic HCV infection; LC: patients with liver cirrhosis; HCC: patients with LC and hepatocellular carcinoma; Number (*n*), not applicable (NA); ^a^: Results expressed as mean ± SD; ^b^: Results expressed as number (% of patients in the corresponding group); ^c^: Results expressed as median (range).

**Table 2:** Correlations of *IL9R* expression with demographic, clinical, and laboratory characteristics of the study patients.

Variable	CHC		CHC-LC		CHC-HCC	
	r/rho	*p* Value	r/rho	*p* Value	r/rho	*p* Value
Age	0.242	0.241	0.187	0.405	0.181	0.387
Serum albumin	−0.436	0.091	0.091	0.560	−0.370	0.063
Serum total bilirubin	0.392	0.134	0.141	0368	0.036	0.861
ALT	0.133	0.804	0.186	0.231	0.186	0.676
AST	0.141	0.879	0.175	0.261	0.157	0.782
ALP	0.198	0.719	−0.064	0.686	0.192	0.346
Prothrombin time	0.324	0.221	0.202	0.194	0.098	0.634
INR	0.111	0.420	0.177	0.261	0.166	0.417
WBC	0.084	0.784	0.085	0.694	0.102	0.697
Lymphocyte	-	-	0.091	0.688	0.224	0.329
Neutrophil	-	-	0.164	0.503	0.175	0.533
Hb	0.587	0.298	−0.156	0.410	−0.025	0.915
HCT	0.571	0.315	−0.089	0.635	−0.176	0.445
Serum creatinine	0.121	0.811	0.068	0.706	0.135	0.630
HCV-RNA	0.311	0.280	0.054	0.791	0.341	0.233
Child-Pugh score	-	-	0.127	0.423	0.308	0.126
MELD	-	-	0.197	0.281	0.081	0.786
AFP	-	-	-	-	0.632	0.368

CHC: patients with chronic HCV infection; LC: patients with liver cirrhosis; HCC: patients with LC and hepatocellular carcinoma; r: Pearson’s correlation coefficient; rho: Spearman’s rank correlation coefficient.
